# Instagram in Histology: Enhancing Medical Education in Brazil

**DOI:** 10.1007/s40670-026-02844-y

**Published:** 2026-08-06

**Authors:** Geovanna Soares de Queiroz, Gustavo Pereira Figueiredo, Maria Eduarda Pequeno da Costa Reis, Guilherme Moreira de Queiroz Coutinho, Gabriel Viliod Vieira, Gleici da Silva Castro Perdoná, Mariana Kiomy Osako, Katiuchia Uzzun Sales

**Affiliations:** 1 Department of Cell and Molecular Biology, Ribeirão Preto Medical School, University of São Paulo, Avenida Bandeirantes 3900, Ribeirão Preto, SP 14049-900 Brazil; 2 Department of Social Medicine, Ribeirao Preto Medical School, University of São Paulo, Ribeirao Preto, Brazil

**Keywords:** Histology education, Undergraduate education, Medical education, Online learning tool, Active learning, Brazilian medical education, Instagram

## Abstract

The exponential expansion of medical knowledge intensifies cognitive load in undergraduate medical education, challenging learning efficiency and long-term retention. While digital learning tools have been widely explored in high-income settings, their role in contexts marked by structural inequalities in access and educational resources remains less understood. In Brazil, disparities in digital infrastructure and access to formal educational support systems create a distinct learning environment in which widely available platforms such as Instagram may represent a scalable and low-cost microlearning strategy. In this study, we evaluated the integration of Instagram into histology teaching within a Brazilian medical school using the Kirkpatrick model. The 2021 cohort of first-year medical students experienced two instructional approaches: traditional instruction during the abdominal histology module and a hybrid approach incorporating Instagram-based microlearning during the pelvic histology module. Student performance was assessed through practical and theoretical examinations, and engagement was measured through structured questionnaires. A multi-cohort comparison revealed that students exposed to Instagram in 2021 achieved significantly higher practical and final grades in pelvic histology compared to cohorts from 2019 to 2022, in which Instagram was not used. Students also reported high levels of satisfaction, citing accessibility, flexibility, and reinforcement of learning as key benefits, despite recognizing the potential for distraction. These findings suggest that, in resource-limited educational settings, social media platforms such as Instagram can serve as effective adjuncts, enhancing engagement and supporting knowledge retention. Importantly, this study highlights the context-dependent value of digital microlearning strategies, particularly in middle-income countries where scalable and accessible educational innovations are critically needed.

**Supplementary Information:**

The online version contains supplementary material available at https://doi.org/10.1007/s40670-026-02844-y.

## Introduction

Mastering histology plays a crucial role in developing competent physicians, as this knowledge fosters critical thinking, enabling the recognition and interpretation of a broad spectrum of pathological conditions [1]. In fact, a study of North American physicians found that 88% considered histology an essential part of the medical curriculum, and 66% reported using their histological knowledge frequently in clinical practice [2]. Nonetheless, unfortunately, medical students often struggle to perceive the practical relevance of histology in their future professional practice. Consequently, modernizing histology education to increase student motivation has become imperative [3].

As a conceptual discipline, the challenges in learning and retaining histology content are substantial. Unlike gross anatomy and physiology, histology requires students to interpret complex two-dimensional microscopic images and mentally reconstruct three-dimensional tissue organization, a process that demands well-developed visual pattern recognition, spatial reasoning skills and either optical or virtual microscopes [4, 5]. Because many students have limited prior exposure to microscopic anatomy, this visual interpretation represents an additional cognitive challenge during the early stages of medical training. Moreover, medical students often struggle to retain previously learned material, frequently forgetting information before they have fully assimilated new content [6]. Indeed, the German psychologist Hermann Ebbinghaus demonstrated that forgetting follows a progressive curve in which knowledge retention steadily declines over time, suggesting that memory effectiveness decreases as the interval since learning increases [6, 7]. To enhance content retention, it is essential to integrate practical, evidence-based learning strategies into the curriculum, as newly acquired conceptual knowledge tends to fade when learned passively [1, 6]. Among the most effective evidence-based strategies are three active learning techniques: the testing effect, free recall, and spaced repetition [7]. The testing effect highlights that repeated testing, combined with immediate feedback, results in significantly better long-term knowledge retention than simply rereading the same material [8]. Free recall, in turn, involves deliberately retrieving previously learned information without external cues, a process that strengthens memory consolidation more effectively than passive review [9]. Finally, spaced repetition, revisiting content at progressively longer intervals, has been shown to enhance both long-term retention and overall academic performance [8]. In this context, incorporating alternative educational tools may promote more innovative and engaging approaches that make learning both more effective and enjoyable [10, 11].

According to Cognitive Load Theory (CLT) [12, 13], motivation plays a crucial role in managing cognitive resources during the learning process. A motivated learner is more likely to invest mental effort, thereby enhancing the germane load, which is the cognitive load directly associated with constructing meaningful understanding and developing expertise. When students perceive histology as relevant and engaging, they are more inclined to dedicate cognitive resources to mastering complex concepts. This active engagement fosters more efficient schema formation, allowing students to integrate new information with prior knowledge more effectively [12, 13]. Over the past decade, smartphones and social media have become increasingly prevalent in higher education as tools to foster student engagement. Moreover, contemporary medical students exhibit advanced digital literacy and routinely engage with technology-mediated educational environments. Therefore, aligning their educational experiences with their technological fluency can optimize engagement and learning outcomes. Indeed, a growing body of evidence underscores the benefits of integrating social media into medical education [14, 15]. For instance, Maske et al. [16] conducted a study involving 250 first-year medical students who participated in a WhatsApp group dedicated to histology instruction. The supervising educator shared digital histological images with annotations and detailed explanations, which were followed by discussions and clarifications. After the examination, students provided overwhelmingly positive feedback, citing improved comprehension and retention, as well as the flexibility to engage with materials at their convenience and from any location.

Despite growing evidence supporting the educational use of social media in high-income and digitally homogeneous settings [17, 18], evidence from middle-income countries marked by digital inequality remains scarce [19]. In many Brazilian medical schools, access to specialized digital resources, such as virtual microscopy platforms, remains limited. Even at our institution, the largest public university in Brazil, virtual microscopy was only fully implemented in 2021 [4]. In contrast, smartphones and social media platforms such as Instagram are already widely available to students, representing a practical and scalable alternative that may help bridge gaps in access to educational resources [20]. Moreover, structured active learning strategies such as retrieval practice and spaced repetition have been insufficiently examined within Instagram-based educational interventions. In light of these gaps, we hypothesized that a structured Instagram-based educational approach would enhance academic performance and promote positive student perceptions of accessibility and engagement within Brazil’s heterogeneous digital landscape. To test this hypothesis, we analyzed three cohorts of first-year medical students enrolled in 2019, 2021, and 2022. We first compared academic performance between the abdominal and pelvic histology modules within the 2021 cohort to assess the effect of introducing Instagram-based microlearning in the same student population. We then compared student performance across cohorts by evaluating the abdominal histology modules (2019, 2021, and 2022), which were all taught using traditional instruction, and the pelvic histology modules, in which only the 2021 cohort received the Instagram-based intervention. The impact of the intervention was evaluated using the Kirkpatrick model, a widely adopted framework for assessing educational outcomes. This model defines four hierarchical levels: learner reaction, learning, behavioral change, and organizational impact [21]. In this study, we assessed Levels 1 and 2. Level 1 captured students’ perceptions of Instagram as a learning tool, whereas Level 2 measured knowledge acquisition through quantitative analysis of histology examination performance.

## Materials and methods

### Participants

One hundred and one first-year medical students (62 men and 39 women) were invited to participate (Table 1). Eligibility criteria included enrollment in the first year of medical school and attendance in both the abdominal histology course, taught using traditional methods, and the pelvic histology course, in which Instagram was incorporated as a complementary educational tool. This design enabled a direct within-cohort comparison under both instructional conditions. To extend the analysis, the academic performance of students from 2019 (60 men and 39 women) and 2022 (49 men and 49 women) (Table 1), who studied pelvic histology without Instagram, was compared with that of the 2021 cohort, which incorporated Instagram as a complementary resource. Abdominal and pelvic histology were selected because they are consecutive modules taught to the same cohort during the same semester. The Instagram intervention was implemented during the pelvic histology module, which occurs later in the course, after students have become familiar with the discipline, laboratory activities, and assessment format. This timing was considered more appropriate for evaluating the acceptance and potential educational impact of Instagram as a complementary learning resource. The 2020 cohort was excluded due to the fully remote teaching format implemented during the COVID-19 pandemic. Students from the 2021 cohort provided written informed consent before participating in the study, which included the Instagram intervention and perception questionnaire. For the 2019 and 2022 cohorts, only fully anonymized academic performance data were retrospectively analyzed. Because no identifiable personal information was collected or accessed, the Institutional Research Ethics Committee approved a waiver of informed consent for these cohorts (Ethics Committee of the University Hospital, Ribeirão Preto Medical School, University of São Paulo, protocol #74309621.1.0000.5440).

**Table 1:** Demographic characteristics of participants

Students’ and participants’ demographics		
Characteristic	Total	
2019 Students		
Sex		
Female, *n* (%)	**39 (40.8)**	
Male, *n* (%)	**60 (59.2)**	
Number of Students; *n* (%)		**99 (100)**
2021 Participants		
Sex		
Female, *n* (%)	**39 (38.6)**	
Male, *n* (%)	**62 (61.4)**	
Number of Participants; *n* (%)		**101 (100)**
2022 Students		
Sex		
Female, *n* (%)	**49 (50)**	
Male, *n* (%)	**49 (50)**	
Number of Students; *n* (%)		**98 (100)**

### Instagram Design and Analysis

An Instagram account named @histoelogico (@histoislogic) was created using a dedicated email and kept public for open access and feedback. The account complemented histology lectures with concise posts and interactive quizzes that provided instant feedback (Supplementary Material, Figures S1-S3). Quizzes included image-based questions to help students identify structures and understand their functions. Students also created memes to illustrate pelvic histology concepts, which they later shared in stories and highlights to reinforce their learning. Content was based on standard histology, physiology, and pathology textbooks [22–25], and images were sourced from the Ribeirão Preto Medical School’s digital collection. Posts were designed in Canva, taking about four hours each, while quizzes required about one hour. To assess student interaction, quiz engagement was analyzed based on the number of responses and the frequency of correct and incorrect answers, providing insight into learning effectiveness through Instagram. Content was published over two weeks: concise posts during the first week and quizzes added in the second. In total, 20 posts and 53 quizzes were shared.

### Evaluation of Students’ Perceptions

In the present study, the questionnaire responses were interpreted within the framework of the Kirkpatrick model for educational evaluation [21]. Specifically, the survey captured Level 1 outcomes (learners’ reactions), including students’ perceptions of accessibility, engagement, and the perceived usefulness of Instagram as a complementary learning tool. Briefly, a validated 26-item qualitative questionnaire previously reported in a peer-reviewed study [4] was used to assess students’ accessibility to and perceptions of Instagram as a teaching tool (see Supplementary Material, Table S1). The survey was administered to students from the 2021 cohort and included questions on Instagram use, accessibility as a learning platform, frequency of access, engagement with the histology profile, and students’ perceptions of its effectiveness in supporting learning. An open-ended question was included to allow participants to provide additional comments about their experience. Descriptive analyses were performed using responses to questions 1–8 and 21–26, whereas responses to questions 9–20 were summed to generate a total perception score ranging from 12 to 60, with higher scores indicating more positive perceptions of Instagram as a learning tool.

### Statistical Analysis of Students’ Performance

Examination grades from 2021 were used to evaluate learning outcomes according to the Kirkpatrick evaluation framework (Level 2, learning) [21]. Grades were compared between abdominal histology (without Instagram) and pelvic histology (with Instagram), both of which were graded on a 0–10 scale. Similar exam formats were used for both topics, with equivalent levels of difficulty. The theoretical examination consisted of 10 equally weighted short-answer questions and was graded directly on a 0–10 scale. The practical examination comprised seven equally weighted image-based questions. To allow direct comparison between the two assessment components, the practical examination score was converted proportionally to a 0–10 scale. Post hoc pairwise comparisons were performed using Dunn’s multiple comparison test with Holm adjustment to assess differences in academic performance between the 2021 pelvic and abdominal histology modules [26, 27]. The same assessment covering abdominal and pelvic histology was administered to first-year medical students in 2019 and again in 2022, when Instagram was not used as a learning tool. Academic performance in the pelvic histology module across the 2019, 2021, and 2022 cohorts was first compared using the Kruskal–Wallis test. When significant differences were detected, post hoc pairwise comparisons were performed using Dunn’s multiple comparison test with Holm adjustment to identify differences between individual cohorts. All statistical analyses were performed using R software version 4.5.2 (R Foundation for Statistical Computing, Vienna, Austria) [28]. A p-value < 0.05 was considered statistically significant for all hypotheses tested.

## Results

### Access and Student Engagement with the Instagram-Based Educational Resource

Students were first asked about their access to the Instagram profile. All participants reported owning a personal cell phone or laptop with internet access beyond the university campus (Table 2). Among them, 91% (80/88) successfully accessed the @histoelogico profile, and none reported technical difficulties. The remaining 9% (8 students) did not engage with the profile, citing unfamiliarity with the platform or a preference for other studying methods. These students were nonetheless included in the analysis to prevent selection bias. All students accessed the Instagram profile from home. Most reported doing so during their leisure time (75%, 60/80), while 23% (18/80) accessed it during study sessions for pelvic histology and 2% (2/80) accessed it during both timeframes. Regarding frequency, 85% (68/80) visited the profile weekly, with 49% (39/80) accessing it more than three times per week. The majority (77/80, 96%) spent up to 15 min per visit, 4% (3/80) spent between 15 and 30 min, and none exceeded 30 min of use.

### Student Preference for Instagram as a Complementary Learning Tool

As shown in Table 2, most students (96%, 77/80) viewed Instagram as an essential complement to traditional teaching, highlighting its accessibility, positive effect on content retention, and contribution to study productivity. Moreover, 86% (69/80) appreciated the humorous approach, stating that the use of memes made learning histology easier or much easier. When comparing the two topics, 86% (69/80) considered Instagram an important or significant tool for improving their performance, while only 13% (11/80) perceived no difference. In terms of accessibility, 81% (65/80) found the traditional method worse or much worse than the Instagram-supported approach, whereas 6% (5/80) still preferred the traditional format. For questions 9–20 of the survey (Supplementary Material, Table S1), scores ranged from 1 to 5, with higher values reflecting more positive perceptions of Instagram as a teaching tool. Possible totals ranged from 12 to 60 points. Students’ scores varied from 17 to 59, with an average of 52, indicating a highly favorable evaluation.

### Perceived Benefits of Instagram as a Complementary Learning Tool

Qualitative analysis of the open-ended responses revealed that students perceived Instagram as a valuable complementary learning tool (Table 3). The most frequently reported strengths included its usefulness for reviewing histology content, making learning more enjoyable and engaging, and providing a dynamic educational experience through quizzes, clinical cases, and interactive activities. Students also highlighted that memes and playful language facilitated content retention and increased engagement with the subject. The main limitations identified were the potential for distraction while using the platform, the perception of increased workload, the superficial treatment of some topics, and the temporary nature of Instagram Stories, which limited opportunities for later review.

**Table 2:** Key quantitative results on the access, impact, and student perception of using an Instagram profile as a supplementary teaching tool (*n* = 80)

Indicator	Key results (*n* = 80)
Access and Profile Use	
Device ownership, internet access	100% owned an internet-enabled device.
location and ease of access	All students (100%) accessed the profile from home, and none (0%) reported difficulties accessing the platform.
Time of use	Most students (75%) accessed the profile during their free time, whereas 22.5% used it during dedicated study sessions and 2.5% reported accessing it during both.
Access frequency	Overall, 85% of students accessed the profile at least weekly, with 48.8% accessing it three or more times per week (including 15% daily) and 36.2% accessing it one to two times per week.
Time spent per access	Most students (96.2%) spent less than 15 min per access, including 51.2% who spent less than 5 min and 45% who spent 5–15 min, whereas only 3.8% spent between 15 and 30 min.
Impact on Learning and Productivity	
Productivity evaluation	Most students (86.3%) rated their use of the Instagram profile as productive (50%) or very productive (36.3%).
Content retention	Overall, 92.5% of students reported that the Instagram profile facilitated retention of pelvic histology content, with 37.5% rating its contribution as facilitating and 55% as greatly facilitating retention.
Impact of humor (memes)	Overall, 86.3% of students believed that humorous content (memes) facilitated their learning, with 36.3% rating its contribution as facilitating and 50% as greatly facilitating learning.
Clearing doubts	Overall, 67.5% of students reported that the tool facilitated asking questions and clarifying doubts, with 36.3% rating its contribution as facilitating and 31.2% as greatly facilitating.
Impact on performance	A total of 86.3% of students considered the Instagram profile to have an important impact on their learning compared with other courses, with 50% reporting an important impact and 36.3% reporting a very important impact.
Perception and Acceptance	
Complement to traditional teaching	Most students (96.3%) believed that using virtual tools to complement traditional teaching was suitable for learning, with 16.3% rating this approach as suitable and 80% as very suitable.
Accessibility (Traditional vs. Instagram)	Most students (81.3%) considered the accessibility of the traditional teaching method to be worse than that of the traditional method supplemented with Instagram, with 72.5% rating it as worse and 8.8% as much worse.
Study preference and motivation	Most students (95%) preferred studying with the Instagram profile, and the same proportion reported feeling motivated to use it as a learning tool.
Maintenance and recommendation	All students (100%) believed that the Instagram profile should be maintained, and nearly all (97.5%) would recommend it to other students.

**Table 3:** Students’ open-ended perceptions of instagram as an educational tool

Students’ open-ended perceptions regarding instagram experiences		
Instagram Strengths (n = 80 )		
Engaging with Instagram proves beneficial for reviewing the subject matter	32 (40%)	“Enhances retention during periods of leisure.”
		“It assists in memorization in a relaxed manner.”
Adds dynamism to the educational experience.	23 (28.8%)	“The availability of practice questions without the pressure of time constraints facilitates my learning process.”
		“Clinical cases and quizzes proved to be highly beneficial.”
It is intriguing, lightweight, and enjoyable.	25 (31.2%)	“I was able to review the content much more frequently and in a comfortable manner.”
		“It seems that the study time is more enjoyable, dynamic, and lighter.”
Memes and playfulness	15 (18.8%)	“It’s a playful and interactive way to approach challenging histology content.”
		“It offers a more enjoyable experience, resembling a “hobby” rather than an arduous effort.”
		“Memes and the utilization of alternative language significantly contribute to the retention of content.”
Increases student engagement with the subject	4 (5%)	“It enabled greater interaction with the subject matter and the professor.”
		“It increases adherence to the course and sparks the class’s interest.”
		“It promotes a deeper understanding of the material, encouraging comprehensive knowledge rather than mere memorization.”
Instagram Weakness ( *n* = 80 )		
Ease of distraction during utilization, impeding the learning process.	6 (7.5%)	“Utilizing my mobile phone diverts my attention from studying.”
Burdens the student with a sense of handling numerous tasks simultaneously.	5 (6.2%)	“The time constraint is substantial, and the volume is overwhelming.”
Treats the subject matter in a more surface-level manner.	3 (3.8%)	“The material presented is less complex than that covered in traditional lectures.”
		“It is not so easy to cover all the content in its full complexity”
The content within stories vanishes, lacking durability over an extended duration.	4 (5%)	“Stories disappeared after a while or polls stopped working after 24 hours, making reviewing difficult.”

### Abdominal Histology Demonstrated Higher Academic Performance Than Pelvic Histology in 2019 and 2022, but Not in 2021

**Fig. 1:**
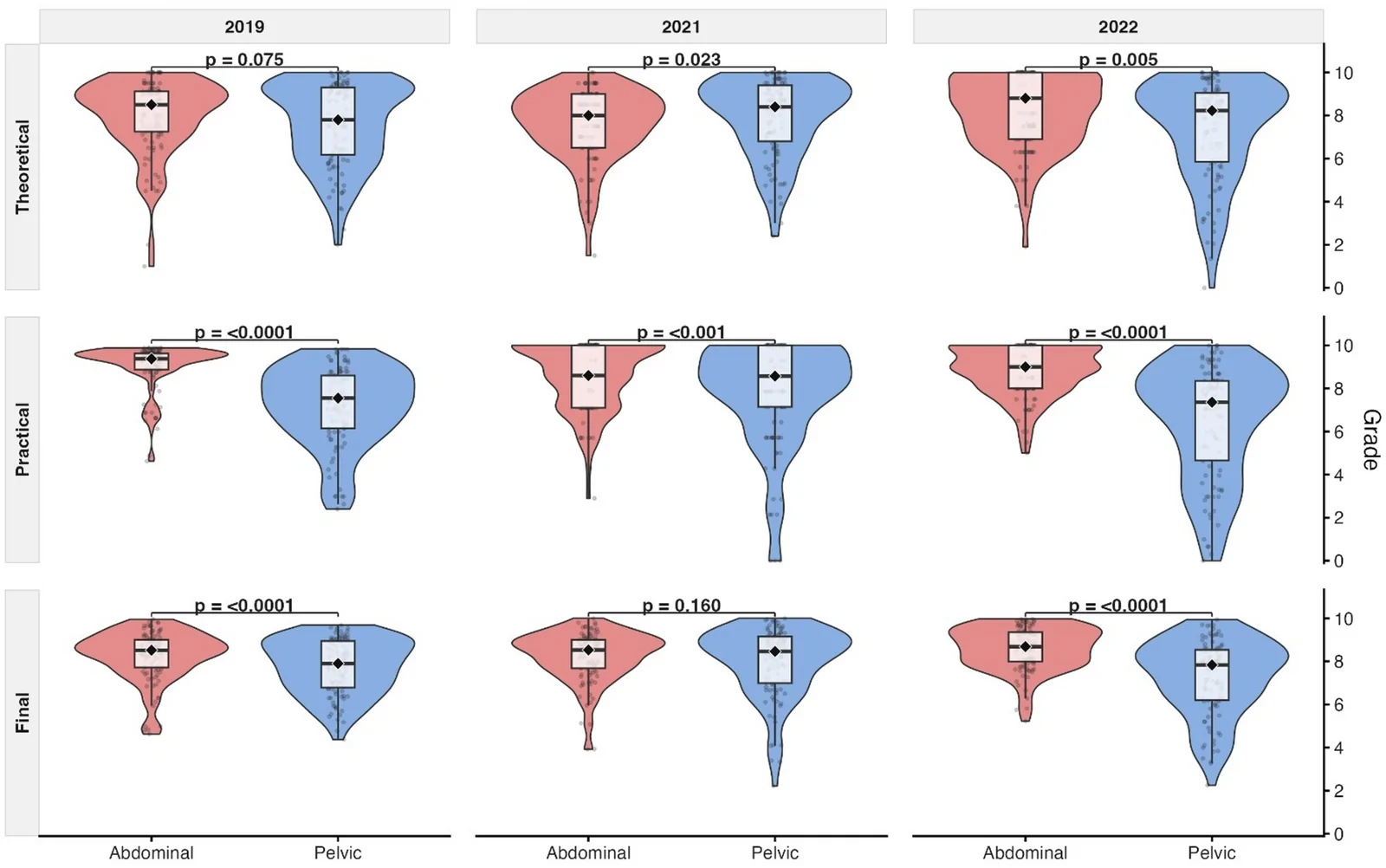
Violin plots showing the distribution of theoretical, practical, and final examination scores in the abdominal and pelvic histology modules. The panels on the left represent the 2019 cohort, the middle panels represent the 2021 cohort, and the panels on the right represent the 2022 cohort. The violin plots illustrate the distribution of individual student scores, with boxplots indicating the median and interquartile range. Comparisons of academic performance across the 2019, 2021, and 2022 cohorts were performed separately for each histology module using the Kruskal–Wallis test followed by Dunn’s multiple comparison test with Holm adjustment when appropriate. Statistical significance is indicated by the p values shown above each comparison

Abdominal histology scores were consistently higher than pelvic histology scores in the 2019 and 2022 cohorts, but not in 2021, as illustrated by the violin plots (Fig. 1) and summarized in Table 4. In 2019, abdominal histology yielded significantly higher practical (median 9.4 vs. 7.6) and final examination scores (median 8.5 vs. 7.9) than pelvic histology (Fig. 1, panels on the left). Although the difference was not statistically significant, theoretical scores also showed a higher median in abdominal histology than in pelvic histology (8.5 vs. 7.8). Similarly, in 2022, abdominal histology demonstrated significantly higher theoretical (median 8.8 vs. 8.2), practical (median 9.0 vs. 7.4), and final examination scores (median 8.7 vs. 7.8) than pelvic histology (Fig. 1, panels on the right). In contrast, the 2021 cohort, in which Instagram was implemented during the pelvic histology module, exhibited a distinct performance pattern. Pelvic histology achieved a significantly higher median theoretical score than abdominal histology (8.4 vs. 8.0), whereas practical and final examination scores showed identical median values between the two modules (8.6 and 8.5, respectively) (Fig. 1, middle panels).

**Table 4:** Academic performance across abdominal and pelvic histology modules by year. Values are presented as median (mean; min-max) for each year

Histology	Assessment	2019	2021	2022	Kruskal–Wallis test (*p*)	Dunn post-hoc comparisons
Abdominal	Theoretical	8.5 (8.0; 1.0–10.0)	8.0 (7.6; 1.5–10.0)	8.8 (8.1; 1.9–10.0)	0.026	2019 vs. 2021 (*p* = 0.026) 2021 vs. 2022 (*p* = 0.023)
Abdominal	Practical	9.4 (9.0; 4.6–9.9)	8.6 (8.7; 2.9–10.0)	9.0 (8.8; 5.0–10.0)	0.864	Not significant
Abdominal	Final	8.5 (8.2; 4.6–9.9)	8.5 (8.2; 3.9–10.0)	8.7 (8.6; 5.2–10.0)	0.042	2019 vs. 2022 (*p* = 0.040) 2021 vs. 2022 (*p* = 0.033)
Pelvic	Theoretical	7.8 (7.6;2.0–10.0)	8.4 (7.9; 2.4–10.0)	8.2 (7.4; 0.0–10.0)	0.318	Not significant
Pelvic	Practical	7.6 (7.2;2.4–9.8)	8.6 (7.7; 0.0–10.0)	7.4 (6.5; 0.0–10.0)	< 0.001	2019 vs. 2021 (*p* = 0.008) 2021 vs. 2022 (p = < 0.001)
Pelvic	Final	7.9 (7.7; 4.4–9.7)	8.5 (8.0; 2.2–10.0)	7.8 (7.2; 2.2–9.9)	0.003	2021 vs. 2022 (*p* = 0.001)

Kruskal-Wallis tests compared the three cohorts within each histology module and assessment type. Dunn post-hoc comparisons were interpreted only when the Kruskal-Wallis test was significant; Dunn p-values were adjusted using Holm’s method

### Comparative Analysis of Pelvic Histology Academic Performance: 2021 vs. 2019 and 2022

Given that abdominal and pelvic histology are distinct modules with different levels of inherent difficulty, academic performance in each module was analyzed separately across the 2019, 2021, and 2022 cohorts (Table 4). Notably, 2021 was the only academic year in which Instagram was incorporated into the teaching strategy of the pelvic histology module. Comparisons of theoretical examination scores in the pelvic module across the three cohorts revealed no statistically significant differences (*p* = 0.318). In contrast, practical examination and final scores remained comparable between the two cohorts that did not use Instagram (2019 and 2022), indicating no significant differences in academic performance in the absence of the intervention. Interestingly, significant differences were observed when the 2021 cohort, in which Instagram was implemented, was compared with both non-Instagram cohorts. Practical examination scores differed significantly between 2019 and 2021 (*p* = 0.008) and between 2021 and 2022 (*p* < 0.001). Likewise, final examination scores were significantly different between 2021 and 2022 (*p* = 0.001). In contrast, comparisons of academic performance in the abdominal histology module across the three cohorts revealed a different pattern. Significant differences were observed in theoretical examination scores between the 2019 and 2021 cohorts (*p* = 0.026) and between the 2021 and 2022 cohorts (*p* = 0.023). Final scores also differed significantly between the 2019 and 2022 cohorts (*p* = 0.040) and between the 2021 and 2022 cohorts (*p* = 0.033). However, unlike the pelvic histology module, no statistically significant differences were found in practical examination scores among the three cohorts (*p* = 0.864).

## Discussion

In the present study, Instagram was evaluated as a complementary, low-cost educational strategy for histology teaching in a Brazilian medical school. Although previous studies have explored the use of Instagram in histology education, particularly in high-income countries such as Spain and the United States [18, 29], our findings demonstrate that this widely used social media platform can also be successfully integrated into medical education in a middle-income country. The novelty of this study lies in its implementation within a setting where access to specialized digital educational resources remains limited, highlighting the potential of readily accessible platforms such as Instagram to expand learning opportunities and complement traditional teaching [30]. Importantly, 96% of students viewed Instagram as an essential complement to traditional teaching, highlighting its accessibility, positive effect on content retention, and contribution to study productivity. These findings are consistent with previous studies from the United States and Spain, which also reported high student acceptance of Instagram as a valuable adjunct to conventional histology instruction [18, 29]. These results reinforce the potential of Instagram as an accessible and effective tool for active learning, in agreement with previous research [29, 30].

Indeed, the Instagram intervention was intentionally designed to incorporate retrieval practice through repeated quiz-based self-assessment with immediate feedback and spaced repetition by distributing educational content and quizzes throughout the module rather than presenting all material in a single session [31]. Our results extended previous findings by comparing the grades of the same participants in abdominal and pelvic histology. Unexpectedly, higher scores in pelvic histology were found in theoretical rather than practical exams. This may be explained by the repeated exposure to both visual and theoretical content on the platform, suggesting that Instagram enhances short-term retention and academic performance in histology. Because significantly higher practical scores were observed in abdominal histology compared with pelvic histology, we conducted an additional analysis focusing exclusively on pelvic histology across cohorts. The 2021 pelvic histology cohort, in which Instagram was implemented, was compared with the 2019 and 2022 cohorts, in which it was not used. Higher scores were observed in 2021 relative to both comparison years. These findings support an association between the use of Instagram and higher academic performance, possibly facilitated by the platform’s engaging visual format [18, 29].

Student satisfaction was another key aspect of this process. Most students expressed a positive perception of Instagram as a learning resource, emphasizing its flexibility, attractiveness, and accessibility. However, some reported that the quizzes were brief and lacked depth, a limitation related to the platform’s format, which favors short and fast posts. Others felt overwhelmed by the constant exposure to study material, suggesting that clear boundaries are necessary to strike a balance between accessibility and well-being. As Garcia and colleagues observed, insufficient teaching time can make histology challenging, and social media can help address this issue when used purposefully [11].

The implementation of Instagram in this context highlights the importance of thoughtfully incorporating technology into educational practice. Although digital platforms can enhance participation and engagement, they are most effective when used to complement, rather than replace, traditional instruction and face-to-face interaction [32]. Appropriate guidance and moderation are essential to ensure that social media supports, rather than overwhelms, the learning process. In the context of educational evaluation, the findings of this study can also be interpreted through the Kirkpatrick model, a widely adopted framework for assessing the impact of educational interventions [33, 34]. According to this model, Level 1 reflects learners’ reactions and satisfaction, whereas Level 2 evaluates knowledge acquisition and learning outcomes [33, 34]. Our results addressed both levels. Students reported highly positive perceptions regarding accessibility, engagement, and usefulness of Instagram as a complementary learning tool, corresponding to Kirkpatrick Level 1 outcomes. Additionally, the improvement observed in pelvic histology examination performance during the year in which Instagram was implemented reflects Level 2 learning outcomes, suggesting that the intervention contributed to measurable knowledge gains. The 2021 cohort displayed a distinct performance pattern compared with the 2019 and 2022 cohorts. Whereas abdominal histology consistently outperformed pelvic histology in the years without Instagram (2019 and 2022), this difference was no longer observed when Instagram was incorporated into the pelvic histology module (2021). Significant improvements in practical examination scores were observed only in the pelvic histology module, whereas abdominal histology scores remained comparable across cohorts. Indeed, the 2021 pelvic histology cohort outperformed the 2019 and 2022 cohorts in practical examination scores, whereas no differences were observed among the corresponding abdominal histology cohorts. Together, these findings provide additional support for the potential educational value of Instagram as a complementary learning tool. Although the observed improvement in test scores was modest, it should be interpreted in the context of a brief, low-cost educational intervention. Even small but statistically significant gains may be educationally meaningful when achieved through an easily scalable strategy that was highly accepted by students in our cohort. Similar applications of the Kirkpatrick framework have been used to evaluate digital and social media–based learning strategies in medical education, demonstrating that positive learner reactions often accompany improved knowledge retention and academic performance [35, 36]. Within this framework, the present findings support the educational value of structured social media interventions as complementary tools in medical curricula. Finally, the relevance of this study lies in its implementation within a middle-income country, where access to specialized digital educational resources, such as virtual microscopy platforms, remains limited [30]. In addition, much of the educational content available on social media is produced in English, which may limit its accessibility for many Brazilian undergraduate students [37, 38]. In this context, delivering structured educational content in Portuguese through a widely used platform such as Instagram represents a practical, low-cost, and scalable strategy to complement traditional histology teaching. However, because we did not directly assess socioeconomic status, internet quality, or differential access to digital resources, our findings should not be interpreted as evidence that Instagram reduces digital inequality. Rather, they demonstrate that a familiar social media platform can be feasibly integrated into an existing medical curriculum to reinforce learning and enhance student engagement.

This study has several limitations. Long-term knowledge retention was not assessed, and the study was not randomized. Furthermore, comparisons across academic cohorts may have been influenced by natural differences in student characteristics, motivation, and academic performance. Differences between cohorts may also reflect year-specific variations in examination difficulty or instructional dynamics despite efforts to maintain comparable teaching and assessment. Therefore, the observed differences should be interpreted with caution, as they may reflect both the effects of the intervention and inherent variability among cohorts. Future research should include multiple cohorts and examine additional digital strategies to assess reproducibility and long-term educational impact.

## Conclusions

In conclusion, the structured implementation of Instagram as a complementary educational strategy demonstrated pedagogical relevance within the context of Brazilian undergraduate medical education. Although no significant difference was observed in overall final grades within the same cohort (abdominal and pelvic histology, 2021), the comparative analysis across years revealed higher academic performance in pelvic histology in 2021, the only year in which Instagram was incorporated. From the perspective of the Kirkpatrick evaluation model, the intervention demonstrated positive outcomes at both Level 1 (learner reaction) and Level 2 (learning). Students reported favorable perceptions of Instagram as an accessible learning tool, while examination results suggested improved knowledge acquisition during the year in which the platform was implemented. These findings reinforce the value of social media–based educational strategies as complementary resources capable of enhancing engagement and supporting measurable learning outcomes in medical education.

Beyond performance metrics, the intervention fostered engagement, accessibility, and flexible content review, elements that are especially meaningful in educational settings characterized by heterogeneous digital access. By incorporating principles of retrieval practice and spaced repetition through interactive quizzes and visually structured content, Instagram provided an environment conducive to active learning while respecting the role of face-to-face instruction.

Importantly, this study contributes novel evidence from a middle-income country with documented digital inequality, expanding the literature beyond predominantly high-income contexts. When thoughtfully implemented and properly moderated, social media platforms may serve not as replacements for traditional teaching but as scalable and inclusive tools capable of enhancing motivation and reinforcing learning. Future multicenter and longitudinal investigations are warranted to assess long-term educational impact and reproducibility across diverse curricular settings.

## Supplementary Information

Below is the link to the electronic supplementary material.

Supplementary Material 1

Supplementary Material 2

## Data Availability

Anonymized datasets generated and analyzed during the current study are available from the corresponding author upon reasonable request, in accordance with institutional and ethical guidelines.
