# Supplementary material for: Instagram in Histology: Enhancing Medical Education in Brazil
Source: Med Sci Educ. 2026 Aug 6;36(6):2819–28. doi: 10.1007/s40670-026-02844-y (PMC13759890; doi:10.1007/s40670-026-02844-y)
Supplement: Supplementary file 1 [file 40670_2026_2844_MOESM1_ESM.docx]

**Supplementary Figure S1** Posts on Instagram’s feed. Example of @histoelogico Instagram feed post regarding pelvic histology content. **A.** The figure exhibits a histological section of the endometrium, briefly explaining progesterone's action during both its early and late secretory phases post-ovulation, besides explaining the cellular aspect and the glycogen accumulation. **B.** The figure displays a histological section of the ovary with its histological division (capsule, cortex and medulla) and summary its function (gametogenesis and steroidogenesis). **C.** The figure presents a schematic diagram illustrating renal vasculature, from the renal artery to the efferent arteriole within the cortex. **D.** The figure contains a histological section of the juxtaglomerular apparatus with a brief explanation of each of its components: macula densa, juxtaglomerular cells and extraglomerular mesangial cells.

**Supplementary Figure S2** Quiz stories on Instagram. Example of a quiz conducted on @histoelogico Instagram Stories regarding pelvic histology content. **A.** The figure displays renal histology with a quiz asking which part of the organ is circled in red, with the options renal pyramid and renal cortex. **B.** The figure features a histological section with a quiz about the corresponding organ, with the options prostate, epididymis, seminal vesicle and bulbourethral gland.

**Supplementary Figure S3** Answers to quiz questions in the story. Example of quizzes conducted via @histoelogico Instagram Stories regarding pelvic histology content. A, B, C, and D. the figures demonstrate student engagement with the quiz to identify the corresponding structure in the histological section; at the bottom of the images the tally of responses for each option is displayed, with the correct answer marked by a green checkmark.
