# Supplementary material for: Instagram in Histology: Enhancing Medical Education in Brazil
Source: Med Sci Educ. 2026 Aug 6;36(6):2819–28. doi: 10.1007/s40670-026-02844-y (PMC13759890; doi:10.1007/s40670-026-02844-y)
Supplement: Supplementary file 2 [file 40670_2026_2844_MOESM2_ESM.docx]

**Supplementary Table S1** Survey Questionnaire on Students' Perceptions of Instagram as a complementary histology teaching tool. The survey was administered to first-year medical students from the 2021 cohort (N = 80) and comprised 26 items. The first column indicates the number of each question. The second column contains the questions asked. The third column indicates the possible responses to each question. The fourth column shows the number of students who selected each response, followed by its percentage.

| **Survey Questionnaire** | | | |
| --- | --- | --- | --- |
| **Question Number** | **Question** | **Possible Answers** | **Student Responses N (%)** |
| **1** | Do you have your own cell phone/notebook? | Yes | 80 (100%) |
|  |  | No | 0 (0%) |
| **2** | Do you have internet access outside of the university? | Yes | 80 (100%) |
|  |  | No | 0 (0%) |
| **3** | Did you access the Instagram profile @histoelogico during the "Abdominal and Pelvic Histology" class? | Yes | 80 (100%) |
|  |  | No | 0 (0%) |
| **4** | Did you have any difficulties accessing the Instagram profile @histoelogico? | Yes | 0 (0%) |
|  |  | No | 80 (100%) |
| **5** | Where did you usually access the Instagram profile @histoelogico? | At home | 80 (100%) |
|  |  | In college | 0 (0%) |
| **6** | When did you usually access the Instagram profile? | During the study time dedicated to the subject | 18 (22.5%) |
|  |  | During the free time | 60 (75%) |
|  |  | Both timeframes | 2 (2.5%) |
| **7** | How often did you access the Instagram profile? | Less than 1 time a month | 3 (3.8%) |
|  |  | Less than 1 time a week | 9 (11.2%) |
|  |  | 1-2 times a week | 29 (36.2%) |
|  |  | 3 or more times a week | 27 (33.8%) |
|  |  | Daily | 12 (15%) |
| **8** | How much time did you spend on each access on average? | Less than 5 minutes | 41 (51.2%) |
|  |  | 5 - 15 minutes | 36 (45%) |
|  |  | 15 - 30 minutes | 3 (3.8%) |
|  |  | 30 - 60 minutes | 0 (0%) |
|  |  | More than 60 minutes | 0 (0%) |
| **9** | How would you rate the difficulty of accessing the Instagram profile @histoelogico? | Very difficult | 2 (2.5%) |
|  |  | Difficult | 0 (0%) |
|  |  | Intermediate | 0 (0%) |
|  |  | Easy | 8 (10%) |
|  |  | Very easy | 70 (87.5%) |
| **10** | How would you rate your productivity when using the Instagram profile? | Very unproductive | 2 (2.5%) |
|  |  | Slightly unproductive | 1 (1.2%) |
|  |  | Indifferent | 8 (10%) |
|  |  | Productive | 40 (50%) |
|  |  | Very productive | 29 (36.3%) |
| **11** | Do you believe that using virtual tools to complement the traditional teaching model (lectures and formative activities), such as the Instagram profile @histoelogico, is suitable for learning? | Not suitable | 1 (1.2%) |
|  |  | Somewhat suitable | 0 (0%) |
|  |  | Indifferent | 2 (2.5%) |
|  |  | Suitable | 13 (16.3%) |
|  |  | Very suitable | 64 (80%) |
| **12** | Do you believe that the Instagram profile @histoelogico facilitated your learning? | Did not facilitate | 1 (1.2%) |
|  |  | Facilitated a little | 1 (1.2%) |
|  |  | Indifferent | 5 (6.3%) |
|  |  | Facilitated | 29 (36.3%) |
|  |  | Facilitated a lot | 44 (55%) |
| **13** | Do you believe that using the Instagram profile @histoelogico facilitated the retention of pelvic histology content? | Did not facilitate | 1 (1.2%) |
|  |  | Facilitated a little | 1 (1.2%) |
|  |  | Indifferent | 4 (5%) |
|  |  | Facilitated | 30 (37.5%) |
|  |  | Facilitated a lot | 44 (55%) |
| **14** | Do you believe that using the Instagram profile @histoelogico facilitated asking and clearing doubts about histology? | Did not facilitate | 2 (2.5%) |
|  |  | Facilitated a little | 2 (2.5%) |
|  |  | Indifferent | 22 (27.5%) |
|  |  | Facilitated | 29 (36.3%) |
|  |  | Facilitated a lot | 25 (31.2%) |
| **15** | Do you believe that the humorous content (memes) involving histology on the Instagram profile @histoelogico facilitated your learning? | Did not facilitate | 2 (2.5%) |
|  |  | Facilitated a little | 4 (5%) |
|  |  | Indifferent | 5 (6.2%) |
|  |  | Facilitated | 29 (36.3%) |
|  |  | Facilitated a lot | 40 (50%) |
| **16** | Did you enjoy learning histology through the Instagram profile @histoelogico? | I hated it | 1 (1.2%) |
|  |  | I didn't like it | 1 (1.2%) |
|  |  | Indifferent | 2 (2.5%) |
|  |  | I liked it | 30 (37.5%) |
|  |  | I liked it very much | 46 (57.5%) |
| **17** | When comparing your performance in the Histology of the Digestive System course with the Histology of the Genitourinary System course, do you believe that the use of the Instagram profile @histoelogico had a significant impact on your learning? | Very unimportant | 1 (1.2%) |
|  |  | Slightly important | 4 (5%) |
|  |  | Indifferent | 6 (7.5%) |
|  |  | Important | 40 (50%) |
|  |  | Very important | 29 (36.3%) |
| **18** | Regarding accessibility, do you consider the traditional teaching method (lectures and formative activities) for studying histology _________ than the traditional teaching method supplemented with a virtual tool like the Instagram profile @histoelogico | Much worse | 7 (8.8%) |
|  |  | Worse | 58 (72.5%) |
|  |  | Indifferent | 10 (12.5%) |
|  |  | Better | 5 (6.2%) |
|  |  | Much better | 0 (0%) |
| **19** | Do you consider the complexity of the content available on the Instagram profile @histoelogico ________ than the content available in the traditional teaching method (lectures and formative activities) | Much smaller | 5 (6.2%) |
|  |  | Smaller | 35 (43.8%) |
|  |  | Indifferent | 35 (43.8%) |
|  |  | Bigger | 5 (6.2%) |
|  |  | Much bigger | 0 (0%) |
| **20** | How important do you consider having the Instagram profile @histoelogico as an additional learning tool? | Very unimportant | 0 (0%) |
|  |  | Slightly important | 1 (1.2%) |
|  |  | Indifferent | 7 (8.8%) |
|  |  | Important | 39 (48.8%) |
|  |  | Very important | 33 (41.2%) |
| **21** | Do you believe that the Instagram profile @histoelogico should be kept as a study tool? | Yes | 80 (100%) |
|  |  | No | 0 (0%) |
| **22** | Would you recommend the Instagram profile @histoelogico as a study tool? | Yes | 78 (97.5%) |
|  |  | No | 2 (2.5%) |
| **23** | Did you feel motivated to use the Instagram profile @histoelogico as a study tool? | Yes | 76 (95%) |
|  |  | No | 4 (5%) |
| **24** | Do you prefer to study with or without the Instagram profile @histoelogico? | With | 76 (95%) |
|  |  | Without | 4 (5%) |
| **25** | Positive aspects in the use of virtual tools, such as the Instagram profile @histoelogico, for the study of histology | Open Question | Open Question |
| **26** | Negative aspects in the use of virtual tools, such as the Instagram profile @histoelogico, for the study of histology | Open Question | Open Question |
